# Papillary Thyroid Carcinoma in a Branchial Cleft Cyst without a Thyroid Primary: Navigating a Diagnostic Dilemma

**DOI:** 10.1155/2013/405342

**Published:** 2013-07-11

**Authors:** Douglas S. Ruhl, Mark F. Sheridan, Joseph C. Sniezek

**Affiliations:** ^1^Department of Otolaryngology-Head & Neck Surgery, Tripler Army Medical Center (TAMC), MCHK-DSH, 1 Jarrett White Road, honolulu, HI 96859, USA; ^2^Tri-State Otolaryngology-Head & Neck Surgery, Huntington, WV 25701, USA

## Abstract

We report a rare case of papillary thyroid carcinoma incidentally found within a branchial cleft cyst. Only four other cases have been described in the literature. A total thyroidectomy and selective neck dissection was performed, and no evidence of occult primary disease was found after review of fine sections. Branchial cleft cysts are the most common lateral neck masses. Ectopic thyroid tissue within a branchial cleft cyst is an unusual phenomenon, and papillary thyroid carcinoma arising from this tissue is extremely rare. Clinicians are left with a diagnostic dilemma when presented with thyroid tissue neoplasm within a neck cyst in the absence of a thyroid primary—is this a case of metastatic disease with a missed primary or rather carcinoma arising in ectopic thyroid tissue? A thorough discussion of the etiologies of these lateral neck masses is reviewed including the embryogenesis of thyroid tissue in a branchial cleft cyst. The prognosis of patients with papillary thyroid carcinoma in lateral neck cysts without a primary site identified appears to be good following excision of the cyst and total thyroidectomy. Other management recommendations regarding these unique lateral neck malignancies are also presented.

## 1. Introduction

Branchial cleft cysts are the most common lateral cystic neck masses. Ectopic thyroid tissue within a branchial cleft cyst is a rare phenomenon, and papillary thyroid carcinoma (PTC) arising from this tissue is extremely rare [1, 2].

We report a case of PTC incidentally found in a branchial cleft cyst. A total thyroidectomy and selective neck dissection was performed, and no evidence of occult primary disease was found after review of fine sections. A brief discussion of the etiologies of these lateral neck masses is reviewed including how ectopic thyroid tissue may be present in a lateral neck mass. Management recommendations regarding lateral neck malignancy without a primary are also presented. This paper was approved by the Tripler Institutional Review Board.

## 2. Case

**Figure 1:**
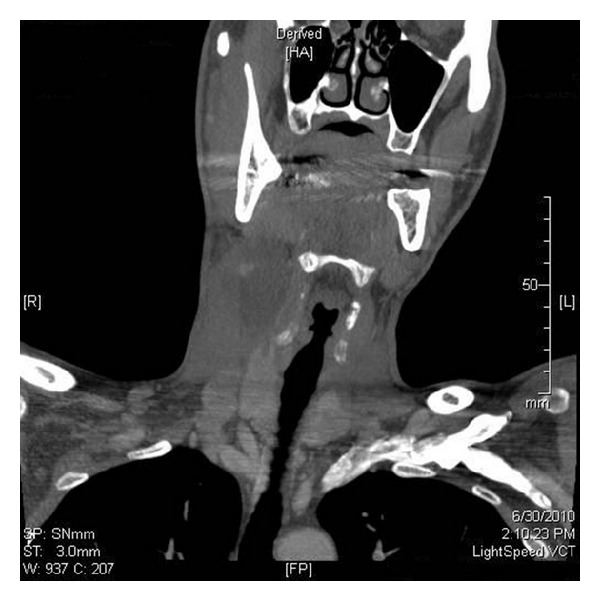
Coronal view of a CT scan with contrast showing right cystic neck mass. Lateral calcification corresponds to the area of malignancy.

**Figure 2:**
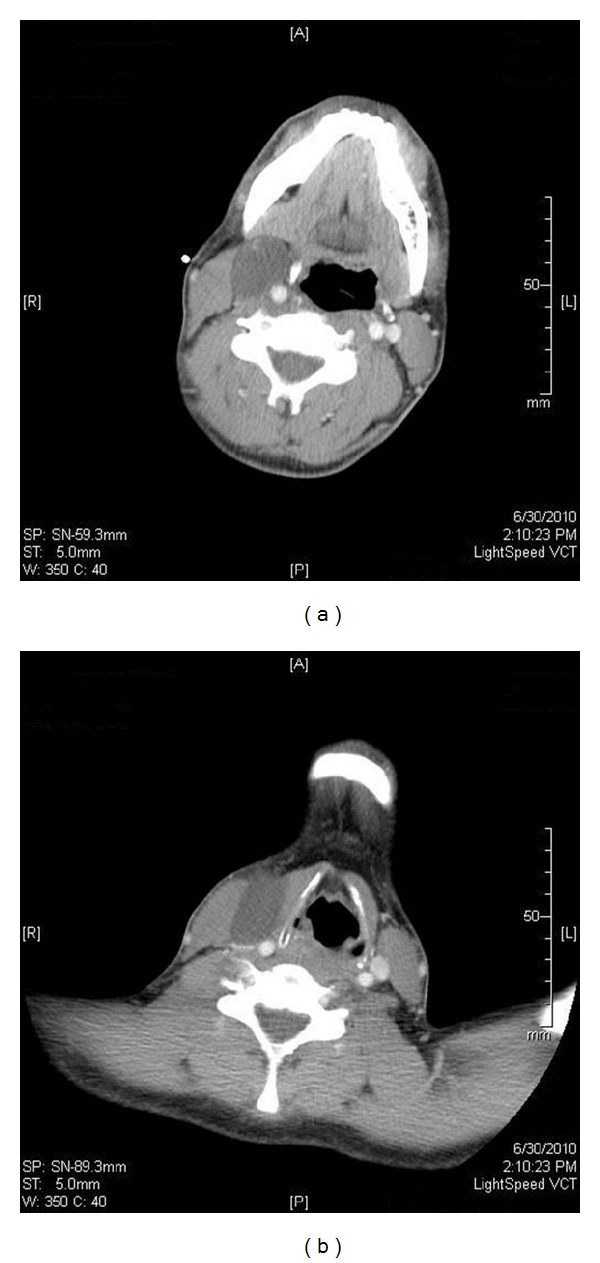
Axial CT Scan with contrast showing the cystic neck mass lateral to the great vessels.

**Figure 3:**
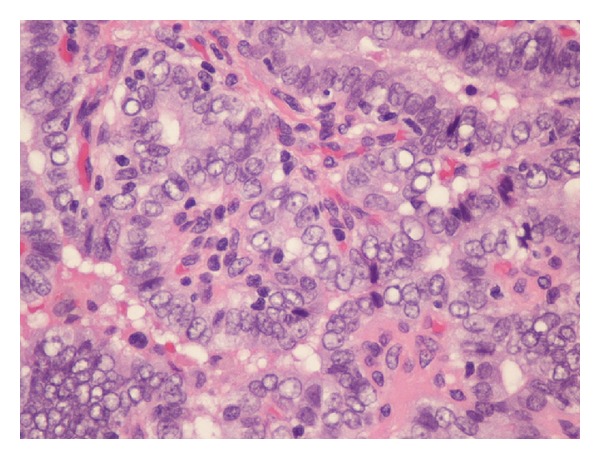
High power (40x) microscopy of the lateral neck mass showing cells with enlarged nuclei, intranuclear cytoplasmic inclusions, nuclear grooves and powdery chromatin which is diagnostic for papillary thyroid carcinoma.

A 20-year-old euthyroid man presented with a history of painless right-sided neck swelling. Physical examination revealed a 6 cm mass in his right neck without a cutaneous fistula. His referring provider ordered a contrast-enhanced CT scan of the neck that showed a cystic lesion in the lateral neck without cervical lymphadenopathy (Figures 1 and 2) consistent with a branchial cyst. Surgical excision was performed, and pathological examination found a 6 cm cystic mass with keratin debris confirming a branchial cleft cyst. Interestingly, there was also a 1 cm focus with diagnostic clusters of PTC within the cyst surrounded by lymphocytic proliferation (Figure 3). There was no pathologic evidence of normal thyroid tissue around the focus of PTC. An ultrasound of the neck was obtained to better evaluate the thyroid which was normal.

Presuming metastatic spread from a thyroid primary, a total thyroidectomy and selective neck dissection (central and ipsilateral) was performed. Serial thin sections of the complete thyroid gland and analysis of lymph nodes did not detect carcinoma. Since the tumor was 1 cm without evidence of other neck diseases, radioactive iodine-131 was not given.

## 3. Discussion

To our knowledge, only four cases have reported PTC arising in a branchial cyst without a primary in the thyroid [1–3, 9]. At least a dozen cases of PTC in lateral neck cysts exist that either found an occult primary in the thyroid or did not pathologically analyze the thyroid. The presence of thyroid carcinoma in a lateral neck cyst is thought to be the result of metastatic spread. This is the obvious conclusion when a thyroid primary is found. However, we are left with a diagnostic dilemma in the absence of a primary—is this metastatic disease with a missed primary carcinoma or rather PTC arising in ectopic thyroid tissue? If it is ectopic tissue in a lateral neck cyst, how did it get there?

Branchial cleft cysts are the most common lateral cystic neck lesion and typically present in the fourth decade of life. However, despite considerable study of branchial cysts, their etiology remains unclear. Previous theories describe branchial cleft cysts as congenital malformations resulting from failure of the branchial pouch apparatus. Recent theories, however, suggest that some of these lateral neck masses are the result of cystic degeneration triggered by epithelial inclusions that migrate into lymph nodes. Proponents of this acquired “inclusion theory” for branchial cyst formation suggest that epithelium from upper aerodigestive tract or glandular tissue enters a cervical lymph node via lymphatics and stimulates degeneration into a lateral cervical cyst [3]. Examination of these lateral cysts demonstrates the presence of lymph tissue [4] making the distinction between cystic lymph nodes and branchial cysts difficult. Some even propose using the term lateral or cervical lymphoepithelial cysts instead since they appear to represent a separate etiology from true branchial cleft cysts that often have cutaneous or pharyngeal fistulas [4, 5]. This cystic change and de novo carcinoma could explain the pathophysiology in our case and has been proposed in similar reports [1, 3].

Another acquired theory proposes that the lateral anlage of the thyroid develops from the fourth-fifth branchial pouch and can aberrantly entrap normal thyroid tissue [6]. Thyroid development from a median anlage via the thyroglossal duct is well described and undisputed. However, lateral contribution via the ultimobranchial body has been shown to contribute parafollicular C cells as well as thyroid glandular tissue [7]. Lateral branchial arch derivatives are implicated in contributing 1–30% of thyroid development [7, 8]. These remnants may be termed thyrocarotid ducts and may manifest as an arrest of lateral migration or as the tubercle of Zuckerkandl if they remain as a protuberance from the thyroid gland [8]. The proximity of branchial arches during development may also explain ectopic tissue.

## 4. Conclusion

Regardless of etiology, the prognosis of patients with carcinoma in lateral neck cysts without a primary site identified after total thyroidectomy appears to be good [1–3, 9]. This might suggest that removing the cyst is therapeutic if it represents de novo carcinoma in ectopic thyroid tissue. It could also represent a missed primary in the thyroid secondary to a missed microcarcinoma, and a thyroidectomy may be considered appropriate treatment.

 Although few examples exist of thyroid carcinomas presenting in a lateral neck cyst without a primary in the thyroid, management recommendations are similar in the literature. Imaging and fine needle aspiration cytology (FNAC) do not take the place of tissue diagnosis, and excisional biopsy should be performed. When the clinician is presented with a thyroid carcinoma in a lateral neck cyst a thorough examination of the neck is necessary. Imaging such as ultrasound and CT scan of the neck plays a role to help distinguish the presence of metastatic nodes from branchial cysts as they can appear similar. Total thyroidectomy is strongly recommended [1–3, 9, 10] and selective neck dissection should be considered [9, 10]. Due to the possibility of papillary microcarcinoma, serial thin sections of all blocks of the totally embedded thyroid should be performed [9]. Adjuvant radioactive iodine might be considered if residual thyroid tissue or disease is suspected [10].
